# Imaginative absorption in schizophrenia: a phenomenological case series and comparison with maladaptive daydreaming

**DOI:** 10.1016/j.ijchp.2026.100729

**Published:** 2026-10-23

**Authors:** Igor J. Pietkiewicz, Radosław Tomalski

**Affiliations:** Research Centre for Trauma and Dissociation, Ignatianum University in Cracow, Kopernika 26, Cracow, 31-501, Poland

**Keywords:** Maladaptive daydreaming, Schizophrenia, Psychosis, Absorption, Mental imagery, Phenomenology

## Abstract

**Background:**

Some individuals diagnosed with schizophrenia report vivid, immersive inner scenarios that may resemble maladaptive daydreaming (MD). However, the organisation, psychological functions, and relationships of such experiences with psychotic phenomena remain poorly understood.

**Aim:**

This study examined the phenomenology and psychological functions of immersive imaginative experiences in individuals with schizophrenia and compared these experiences with published phenomenological descriptions of MD.

**Method:**

A theory-driven comparative case-series design was used. Three individuals with schizophrenia reporting immersive fantasy experiences participated in semi-structured clinical interviews. Cases were analysed across dimensions of agency and control, reality attribution and perceptual qualities, psychological function, and relationships with psychotic phenomena, and then compared across cases and with published descriptions of MD.

**Results:**

Prolonged, immersive fantasies differed substantially in their organisation. Patrick and Kate showed limited agency over intrusive, often distressing imaginative content that could unfold autonomously and become intertwined with hallucinations and delusional interpretations. Dominic retained authorship and awareness of the imagined nature of his technical visualisations but had difficulty regulating and disengaging from them. Across cases, content reflected emotionally salient concerns and different psychological functions, including the expression of conflict and emotional compensation. Features overlapping with MD included prolonged absorption and difficulty regulating engagement, whereas reduced agency over content and believing that imagined interactions or communications were genuinely occurring more clearly distinguished Patrick’s and Kate’s experiences.

**Conclusions:**

Similarities with MD may coexist with marked differences in agency, reality attribution, and relationships with other psychopathological phenomena, suggesting that their diagnostic significance depends on how these features are configured.

## Introduction

Maladaptive daydreaming (MD) is characterised by compulsive, immersive fantasising that can occupy many hours per day and displace academic, occupational, and social engagement (Bigelsen et al., 2016; Pietkiewicz et al., 2018; Somer, 2002). Individuals report vivid, plot-driven scenarios accompanied by strong affect and frequently describe daydreaming as a means of regulating distress, loneliness, shame, or low self-worth (Pietkiewicz et al., 2018, 2023a; Soffer-Dudek et al., 2025). When prolonged and difficult to control, imaginative activity may increasingly take priority over real-life engagement and contribute to social and functional impairment (Pietkiewicz et al., 2023b, 2025; Somer et al., 2017).

Existing descriptions of MD indicate that individuals generally distinguish their daydreams from external reality, even when these experiences are highly vivid and immersive (Mansuklal et al., 2025). Phenomenological work nevertheless describes a profound sense of presence within a created fantasy of a parallel reality (Somer et al., 2016), suggesting that experiential realness should not be equated with reality attribution. A related issue is source monitoring, which concerns judgements about the origin of mental experiences. Source-monitoring errors are well documented in schizophrenia-spectrum disorders, particularly in relation to hallucinations (Damiani et al., 2022). Recent reports have described instances in which individuals with MD later confused imagined events with autobiographical experiences (Somer, 2024; Somer & Otgaar, 2025). However, Meidan and Soffer-Dudek (2026) found no corresponding impairment in an experimental task requiring discrimination between performed and imagined actions. These findings suggest that reports of fantasy – memory confusion in MD do not necessarily indicate a generalised difficulty distinguishing imagination from reality.

A complementary phenomenological perspective is provided by Rasmussen et al. (2018), who developed the Examination of Anomalous Fantasy and Imagination (EAFI) to investigate anomalous imagination in schizophrenia-spectrum disorders. The EAFI distinguishes several dimensions of imaginative experience. *Perceptualisation* (or *spatialisation*) refers to imagery acquiring quasi-perceptual characteristics, including spatiotemporal constancy, explorability, and multimodal qualities. *Autonomisation* refers to imagery developing its own flow or sequence relatively independently of the person's will, although it may still be initiated, explored, or influenced to some extent by the individual. *Erosion of irreality* refers to a weakening of the normally immediate awareness of an experience as imagined, ranging from transient uncertainty requiring reflective checking to more pronounced difficulty distinguishing imagination from memory or perception. These anomalies of imagination are not intrinsically psychotic, although they may become intertwined with hallucinations and delusional experiences (Rasmussen, 2024). In a study comparing clinical and non-clinical groups (N = 81), Rasmussen et al. (2022) found that anomalies of imagination assessed with the EAFI were substantially more frequent in individuals with schizophrenia-spectrum disorders than in those with non-psychotic disorders or healthy controls. However, their diagnostic specificity requires further investigation. Vivid and “as-if-real” experiences described in MD raise the possibility that some of these phenomenological characteristics may also occur outside psychotic disorders, although their configuration and relationship to reality attribution may differ.

Existing accounts of MD distinguish it from psychotic phenomena by preserved awareness that daydreams are self-generated and diverge from external reality; MD itself is not characterised by psychotic delusions or hallucinations (Somer et al., 2016; Soffer-Dudek et al., 2025). However, this distinction has received limited direct phenomenological examination, and it remains unclear whether broad concepts such as insight or reality testing adequately capture the boundaries between MD and psychosis-related imaginative experience. Research on MD has focused on describing the experiences and psychological functions of immersive daydreaming, whereas phenomenological studies of schizophrenia-spectrum disorders have examined alterations in the structure of imagination and self-experience. Little is therefore known about how MD-like imaginative experiences are organised when they occur in the context of psychosis, or about their psychological functions in this context. Direct comparison may help clarify both their phenomenological boundaries and the extent to which their functions overlap.

Accordingly, this paper aims to characterise the phenomenology and psychological functions of immersive imaginative experiences occurring in schizophrenia, with particular attention to their relationships with delusions and hallucinations. By comparing these experiences across cases and with established descriptions of MD, we seek to clarify points of convergence and divergence and contribute to a broader understanding of what we refer to here as the “pathology of fantasy” – clinically significant forms of imaginative involvement across diagnostic contexts.

## Method

### Design

This study employed a comparative case-series design to examine imaginative absorption occurring in schizophrenia and its relationship to MD. A case-series approach was selected because it permits systematic comparison while retaining the contextual and phenomenological richness of idiographic data, which is particularly valuable when exploring complex and under-theorised clinical phenomena (Yin, 2018).

#### Case selection

Participants were selected using purposive, relevance-based sampling. Inclusion criteria were: (1) a clinical diagnosis of schizophrenia; (2) a history of pronounced absorption in fantasy; and (3) the ability and willingness to describe these experiences in detail. The criterion of pronounced absorption in fantasy was not restricted to elaborate narrative daydreaming, as the study sought to examine the phenomenological diversity of immersive imaginative experiences in schizophrenia. Dominic met this criterion through prolonged and absorbing technical visualisations, despite their lack of an elaborate narrative structure. All participants had undergone psychiatric hospitalisation, during which schizophrenia was diagnosed following comprehensive psychiatric assessment according to ICD-10 criteria. They subsequently received long-term outpatient treatment under the care of the second author, a psychiatrist who regularly assessed their symptoms and functioning and monitored their response to pharmacotherapy. Longitudinal clinical observation and available medical records provided further support for these diagnoses and allowed evaluation of their clinical course. At the time of data collection, two participants were in symptomatic remission from psychosis, whereas Kate continued to experience psychotic symptoms but had sufficient insight and verbal capacity to reflect on past and present experiences.

#### Data sources

Primary data consisted of semi-structured interviews conducted by the first author, an experienced clinical psychotherapist, supervisor, and researcher. Interviews lasted 75 min for Patrick, 60 min for Dominic, and 120 min for Kate. They explored the onset and course of psychotic symptoms; the structure and content of imaginative experiences; affective involvement and distress; agency and control; co-occurring hallucinations, delusional beliefs, and explanatory models; and treatment history and recovery. Participants had been in long-term outpatient treatment with the second author, a psychiatrist, psychotherapist, and supervisor, providing access to longitudinal clinical information and facilitating detailed discussion of their experiences. All participants were clinically stable enough to participate in the interviews. Interview data were supplemented by relevant clinical documentation. Interviews were transcribed verbatim, pseudonyms were assigned, and potentially identifying information was removed. Participants provided informed consent for the use of their anonymised narratives for research. The study formed part of the project “Between fantasy and reality: epidemiology and psychopathology of MD,” approved by the Ethics Committee of Ignatianum University in Cracow on 11 July 2025 (no. 19/2025).

#### Analytic approach

A structured, dimension-focused analysis was conducted. Both authors independently reviewed and analysed the interview transcripts, initially examining each case idiographically. Particular attention was given to the organisation and content of imaginative experience, agency and control, perceptual qualities and experiential realness, reality attribution, psychological function, and relationships with psychotic phenomena. These analytic domains were informed by existing research on MD and phenomenological studies of anomalous imagination in schizophrenia. The authors then compared their interpretations and discussed differences until a shared understanding of each case and the relevant analytic dimensions was reached. Case vignettes were developed as syntheses of the interview and clinical material, incorporating selected verbatim quotations to preserve participants’ descriptions of their experiences. Cross-case comparison was subsequently undertaken to identify converging and diverging patterns across the analytic dimensions. The analysis was exploratory and theory-building; no claim of thematic saturation is made.

## Case vignettes

### Vignette: Patrick

#### Patient background

Patrick is a man in his early forties with longstanding schizophrenia and a history of severe polysubstance use beginning in adolescence. He started using cannabis at age 14 and soon progressed to amphetamines, LSD, and psilocybin – sometimes taking “three, four, even five blotters at once.” He used cannabis daily and stimulants intermittently and had undergone several episodes of substance use treatment by age 18. Over the following decade, he experienced repeated psychiatric hospitalisations and participated in several dual-diagnosis treatment programmes, including long-term residential rehabilitation. After achieving sustained abstinence and stabilising on pharmacological treatment, including olanzapine, aripiprazole, and escitalopram, he resumed work as a driving instructor, maintained stable relationships, and remained clinically stable for several years before the interview.

#### Onset and development of symptoms

Patrick traced the onset of his psychotic symptoms to age 17–18. While walking through his neighbourhood, he experienced a sudden, overwhelming internal sensation: “suddenly this dark wave hit me from the back of my head.” He immediately interpreted it as an assault: “I thought my colleagues from the neighbourhood were attacking my soul in an invisible spiritual world.” Although no one was present, he vividly sensed their presence - “I felt they were right next to me”—and imagined them rejecting him: “it felt like they were saying: you are different, you don’t belong here, you’re not one of us anymore.”

He described this moment as the awakening of “another world inside me.” What began as a discrete episode quickly became a pervasive inner environment: “later, even when I wasn’t taking drugs, it became everyday life… from morning until night something would appear.” The experiences occurred irrespective of intoxication: “whether I was sober, on drugs, or drinking—it was always there.” Even during later periods of remission, the scenarios could suddenly reactivate: “I was functioning normally in the real world, and then suddenly that imaginary world appeared again on its own.”

#### Phenomenology of immersive experiences

Patrick’s experiences took the form of involuntary, vivid inner scenarios involving familiar and unfamiliar figures who communicated with him through intrusive thoughts. He repeatedly emphasised that he never heard voices aloud: “I didn’t hear them with my ears – these were thoughts, but they felt like they came from outside me.”

From the beginning, the core theme was interpersonal rejection. Imagined acquaintances from his neighbourhood confronted him with accusations of exclusion. These messages carried emotional weight: “I didn’t want to be rejected—they were my everyday friends,” and he believed these interactions revealed real interpersonal truths: “if they said it in that spiritual world, I thought it must be the same in reality.”

As the illness progressed, the inner world expanded beyond his peers. Characters from television—actors, presenters, politicians, even presidents—began to appear in his mind as real agents rather than merely imagined figures. Watching TV became a potent trigger: “I had the impression that the people on the news knew about me… they came out of the TV and asked what I wanted from them and why I was bothering them.” At the time he believed these encounters were real, although he did not literally see figures emerging from the screen with his eyes. Instead, he experienced vivid, intrusive imagery accompanied by the conviction that the events were happening: “I didn’t see it with my eyes—it was in my imagination, but I thought it was real.” Even minor gestures took on personal significance: “I would walk past the TV and imagine brushing against someone on the screen with my elbow… and then think he knew it was me and would come to ask why I touched him.”

These encounters shared a stable narrative structure: accusation, confrontation, and exclusion. Characters questioned him (“why did you come here—what do you want?”), criticised him as strange or inferior, or told him to leave. The experiences were also accompanied by tactile misperceptions. Patrick felt brief sensations of being touched—“as if someone brushed my shoulder or arm”—which triggered defensive reactions: “I curled up to avoid being touched.” These bodily sensations intensified his sense of intrusion and strengthened his conviction that he was interacting with real agents beyond his own imagination.

A notable shift occurred when he became more engaged in religion and spirituality, partly through rehabilitation programmes. His experiences reorganised into a moral and spiritual cosmology populated by “good and bad souls.” He believed malevolent souls pursued him: “the bad souls were hunting the good ones, and I wanted to be a good soul—so they went after me.” Around the same time, his spiritual concerns merged with delusional grandiosity. He recalled believing he was destined for an elevated religious role - “I thought I would become pope” – which briefly gave his suffering a sense of significance and mission. This spiritual framework organised the perceived hostility within a broader moral narrative of good and evil and connected it with his efforts at moral self-improvement.

These internal scenarios profoundly affected his functioning. Patrick described prolonged episodes of absorption: “I would go silent for a minute, two, five… I was physically with my friends but talking to someone in my head.” He became unable to attend school because he imagined that classmates would torment him: “even when I wasn’t at school, the people from school were in my head.” The experiences increasingly occupied his attention and interfered with his ability to remain engaged with people and activities in his immediate environment.

#### Affective content

The emotional tone of the experiences was consistently and intensely negative. Patrick described “strong fear, very strong fear,” noting that “there were no positive or friendly experiences—almost all were negative.” Persistent internal harassment led to hopelessness and, at times, suicidal ideation.

#### Agency and control

Patrick reported little control over the onset or content of the scenarios. “They just appeared,” he explained. He could not influence what the figures said: “I had no influence over what they told me.”

Early attempts to manage the experiences involved confronting or dismissing the imagined figures: “I told them: go away, leave me alone, what do you want?” but these efforts were ineffective. A turning point came when his psychiatrist encouraged him to postpone internal interactions, a strategy he implemented literally: “I told them: I can’t talk now—come back at nine in the evening.” To his surprise, “they didn’t show up at all,” giving him his first partial sense of influence over the experiences.

At later stages, with symptoms fluctuating, he sometimes checked whether the experiences would reappear: “I would think about my father to see if the whole thing would appear again.” These tests occasionally triggered new episodes and heightened his fear that the experiences could return unexpectedly. He expressed ongoing vulnerability: “once it started, it went its own way.” The scheduling technique—postponing encounters to a later time—provided one of the few experiences of partial control.

#### Reality attribution

Patrick attributed reality to his psychotic experiences: “I thought it was the truth… you can’t just sit down and say: hey, this is fake—and it disappears.” His interpretation of the experiences changed gradually with medication and therapeutic work: “when I entered into contact with them, I sometimes suddenly realised: wait, this is fiction.” He also described some degree of uncertainty even earlier in the illness: “deep down I knew it wasn’t real… I was ashamed to talk about something that doesn’t exist.” In remission, he consistently recognises the experiences as symptoms of his illness, although he occasionally experiences residual anxiety when recalling their intensity.

#### Treatment course and current status

Patrick’s recovery unfolded across multiple detoxifications, psychiatric admissions, and several treatment programmes. A meaningful improvement followed optimisation of his antipsychotic treatment, with psychotic symptoms decreasing even before he achieved sustained abstinence. He subsequently completed a 10-month residential dual-diagnosis programme involving structured therapy, psychoeducation, and medication management. After an earlier relapse following medication discontinuation, he resumed treatment and has remained adherent since. At the time of the interview, he reported complete remission of the immersive experiences: “the inner world is completely gone.” He maintained long-term abstinence and stable occupational functioning and consistently recognised his past experiences as illness-related rather than as genuine interactions or events.

### Vignette: Dominic

#### Patient background

Dominic is a 41-year-old single man with a diagnosis of schizophrenia, currently working as a security guard. He lives with his parents and has an older brother who lives independently. He completed a technical university degree in mechanics and previously attended a secondary school specialising in electronics and industrial automation. Despite this training, he feels he “didn’t learn anything” and experienced his educational path as imposed rather than chosen: “I mixed up the fields… they told me I should go in that direction, and it just went that way.” He never worked in his trained profession and believes that if he had, he would have “only had more failures.”

From childhood he remembers being “a bit different.” In primary school he often lay listening to music, rocking back and forth, and his mother wondered whether he might have “some form of autism.” He recalls stress-related tics, such as twisting his fingers, and chronic difficulty fitting in: “I always stood out from the group.” In social situations he felt clumsy and inadequate, later judging his own utterances harshly: “Most of what I said were stupid things… in technical school I just talked nonsense.” These longstanding social and interpersonal difficulties preceded his later withdrawal and onset of psychiatric symptoms.

#### Onset and development of symptoms

Dominic dates the onset of his psychiatric difficulties to the later years of university. He began withdrawing from social life and spending long hours alone at his computer. “There were three months when I spent all the time with pornography,” he recalls. He would return from classes, stay at home, download films from the internet, and burn them onto CDs, eventually amassing “a whole pile” of discs with videos of women he liked. During this period, he almost stopped leaving the house, and his academic functioning deteriorated. He failed courses, had to repeat a semester, and delayed completing his thesis.

At the same time, he developed increasing anxiety and ideas of reference. He feared that if others learned how much pornography he watched, they would ridicule him and “set people on” him. Crowded situations triggered intense unease: “In the bus, when there were too many people, or in gatherings, the anxiety started.” He felt watched and mocked - “it’s like someone is observing you and constantly making fun of you”—and attempted to avoid being noticed: “I tried to escape people’s eyes.”

After this period of isolation and functional decline, he was diagnosed with schizophrenia and began psychiatric treatment. Over time, his previously broader and more playful imaginative activity progressively narrowed and became increasingly focused on one domain: designing weapons.

#### Phenomenology of immersive experiences

Dominic distinguishes between his earlier “ordinary fantasies” and the more focused, repetitive imaginative activity that developed later. As a younger man he saw himself as “a dreamer.” He imagined becoming a film director, an actor, or someone training martial arts and improving his fitness. These were brief wishes rather than extended plots: “I didn’t create long stories… it was more like a desire that maybe I would sign up for some martial arts, to build my condition.”

After university, in the context of illness and increasing isolation, his imaginative activity became progressively focused on firearms and technical design. He described a turning point: “After I finished studies it got into my head that I have to be somebody, a respected person… that it would be best if I did something outstanding.” This aspiration became increasingly associated with weapons: “It started to focus on firearms,” which he linked to media portrayals of arms designers and dealers as powerful or important. He emphasised that his interest was not directed toward violence: “I wasn’t thinking about killing people… I wanted to be someone respected, maybe that people would be a bit afraid because I know about weapons.”

At its most intense, Dominic’s imaginative activity consisted largely of prolonged technical visualisation of mechanical systems. He spent hours visualising “how the trigger mechanism works, how the parts overlap, how the bolt moves, how the gases are vented, what the ballistics are.” Over the years, he imagined new solutions “so as not to copy old things, but invent something new.” He studied the history of weapons and felt both pride and frustration when he later saw similar mechanisms implemented: “I see that some now come up with similar ideas and they work… my ideas are good, but someone else did them.”

Although the focus is technical, occasional self-relevant elements also appear. He sometimes imagines working in a factory, having his ideas recognised, or running a small company: “I imagine I work in a firm that produces firearms… or that I could set up a small firm, hire some smart people, and lead the team.” These scenes do not take the form of elaborate narratives but rather brief images of possible future roles and achievements. At the same time, he recognises that he lacks the knowledge and practical skills needed to design or manufacture weapons—“I finished a technical school, but I don’t know anything”—and imagines relying on others with greater expertise: “I would need a manager, because you have to know this stuff.” These self-relevant images centre on competence, recognition, and occupational achievement. However, he emphasises that the dominant mental activity is technical problem-solving rather than role-play or extended narrative fantasy: “Mostly I solve technical problems, not imagine long scenes with people.”

#### Affective content

Dominic’s imaginative activity was closely intertwined with loneliness, low self-worth, and a desire to feel competent and recognised. He described it as initially having a compensatory function: “I tried to build myself up through these dreams… that I would escape from this flood of fear of people.” When he had few social contacts and felt inadequate in interpersonal situations, mentally constructing weapons provided a temporary sense of competence and value. The designs represented technical mastery and elevated status; as he put it, people who construct weapons “are more valued.” In this earlier phase, the activity also provided temporary relief from fear and isolation: “When you live a life like this, it’s pleasant in that moment, because you engage in something in your mind.”

Over time, however, the same activity became increasingly frustrating. Although the imaginative process itself could remain pleasurable, prolonged engagement also confronted him with the discrepancy between his imagined achievements and his actual circumstances: “It’s pleasant, but when you do it for hours and it doesn’t translate into reality, it becomes tiring and irritating… there are the urges but not the skills.” Thinking about constructing weapons reminded him of missed educational opportunities and his perceived lack of practical competence. As he became increasingly aware of these discrepancies, an activity that had previously provided relief also highlighted what he regarded as personal failures. “I finished technical school, but I don’t know anything… I should at least know the basics to draw something,” he said, expressing regret about years he felt were lost: “If I had spent those years on studying, I would know something now.”

The affective quality of the activity was therefore ambivalent. It could provide temporary engagement and a sense of imagined competence while also intensifying feelings of inadequacy, regret, and frustration.

#### Agency and control

Dominic retained authorship over his imaginative activity, although individual ideas could arise spontaneously and become difficult to disengage from once he began elaborating them. “These thoughts come on their own… and the ideas give pleasure when you solve them.” At the height of this activity, he could spend “several hours a day” mentally designing weapons, often continuing for much longer than intended. He described difficulty regulating the duration of these episodes: “It was hard to control.”

A notable consequence was sleep disruption. When the activity intensified, his mind could remain occupied with technical problems late into the night: “It lasted for many hours… and even sleep was impossible.” Attempts to disengage were often ineffective: “When you want to go to sleep and you think about it, you can’t fall asleep.” External interruptions could temporarily break the sequence—“my mother would come and say: go do something”—but he often returned shortly afterwards: “I would do something for a moment and then go back to imagining this.”

#### Reality attribution

Dominic consistently recognised his technical visualisations as self-generated and imaginary. He denied ever experiencing them as actual events or communications. When asked whether the imagined scenarios had ever felt as though they were genuinely occurring, he stated that this had never been the case. Throughout the interview, he described his weapon-related mental activity as technical visualisation rather than the enactment of imagined scenes.

His preserved reality attribution regarding these visualisations coexisted with self-referential ideas in other areas of experience. He feared that people would discover his pornography use and “start making fun” or “set people on” him, and in crowded situations he felt watched and ridiculed. He also acknowledged that some experiences of rejection had occurred before the onset of illness, noting that people had “distanced themselves” and “made fun” of him.

#### Treatment course and current status

Dominic began psychiatric treatment after university, when his mother arranged a consultation with a psychiatrist. He later sought help independently and underwent a voluntary psychiatric admission lasting approximately three weeks. He recalled being heavily sedated during hospitalisation—“I mostly just lay in bed… I didn’t think much”—and experiencing the unfamiliar environment as monotonous and unstimulating. He subsequently continued outpatient psychiatric treatment with antipsychotic medication. He felt that treatment had reduced both his prolonged imaginative activity and his pornography use—“The meds handle it”—although he reported fluctuations in symptom intensity over time.

Dominic reported that, despite having taken antipsychotic medication consistently, a marked reduction in his prolonged imaginative activity occurred after the introduction of lurasidone. He estimated that the previously absorbing episodes decreased to “a few minutes maybe per day” and no longer dominated his functioning. This change was accompanied, however, by prolonged periods of markedly reduced mental activity. He described these as periods of “not thinking about anything,” adding, “I have periods when I don’t feel like thinking about anything,” which he experienced not as relief but as a loss of mental activity and initiative. After lurasidone was discontinued at his request, he reported an increase in his imaginative activity.

At present, Dominic works shifts as a security guard and spends much of his free time listening to music at home or at work. He has one friend whom he meets once or twice a month, which he says “is enough,” although he also states that he would prefer to be “more contactable.” He reports chronic difficulties with motivation, organisation, and following through on interests. He regrets not pursuing drawing or computer graphics, which he now regards as more suited to his abilities and temperament. He remains uncertain about his diagnosis, wondering whether it is “really schizophrenia or some kind of schizoid personality,” although he accepts that he has a chronic psychiatric condition affecting his functioning.

He continues to think about weapons briefly each day and sometimes imagines working in an arms-related company or sharing his ideas for money, but these episodes are shorter and less absorbing than in the past. He remains aware that his ideas are unlikely to be realised. Imaginative activity nevertheless continues to provide him with a transient sense of engagement and possibility.

### Vignette: Kate

#### Patient background

Kate is a woman in her early forties with a diagnosis of schizophrenia who has received continuous psychiatric treatment since 2012. She works as a physiotherapist with patients recovering from stroke, often providing home-based rehabilitation. Her work involves emotionally charged encounters, sometimes with patients whose behaviour she experiences as inappropriate, demanding, or morally troubling. Interpersonal stress in these situations is often associated with an intensification of her psychotic experiences. She has a long history of auditory hallucinations and vivid inner imagery, which have persisted, with fluctuating intensity, for more than a decade. Despite these symptoms, she remains employed, engaged in psychiatric treatment, and able to describe her experiences in detail.

#### Onset and development of symptoms

Kate traced the onset of psychotic symptoms to her early twenties, during a period when she was living and working abroad. She recalled episodes of intense fear that someone was entering her apartment and soon began experiencing voices and personally significant communications in her surroundings. “I thought they were filming me on television… the voices were coming from the TV and telling me strange things.” She also experienced birds as communicating different messages to her through their calls. These experiences felt real and personally directed, contributing to a sense that she was “famous” or being monitored.

In the early years, she heard a large, shifting multitude of voices: men, women, children, and animals. They seemed to arise from ordinary environments—“I left the doctor’s office and it felt like the doctor was speaking to me”—and frequently commented on her competence and worth: “they said they didn’t like me… that I wasn’t good at what I do.” At that time, she believed these were real people speaking: “I trusted that the people were real and that they were telling the truth about me… I couldn’t tell the difference.”

Over time, the multitude of voices narrowed to two recurrent male voices, which Kate identified with a psychiatrist and a dentist. The psychiatrist-associated voice was predominantly violent, punitive, and hostile, whereas the dentist-associated voice was supportive, reassuring, and protective. These voices were accompanied by detailed internal imagery involving the same figures. The psychiatrist-associated voice told her she was worthless—“you look like a clown”—and issued threats such as “you should kill yourself.” The dentist-associated voice provided a contrasting commentary, encouraging her and defending her from the attacks: “he tells me I’m smart, that I’ve achieved a lot, that I shouldn’t listen to the other voice… that I should believe in myself.”

The figures in her imagination representing these voices also interact with one another. They argue, insult each other, and engage in violent confrontations: “They kill each other all the time.” Kate described these scenes as vividly imagined and highly immediate, yet clearly located within her internal experience rather than external space. She experienced the interactions as unfolding images in her mind rather than as visually hallucinated figures in the environment.

Her psychosis also included delusional beliefs involving telepathy, thought broadcasting, and being spiritually chosen or exceptional. She believed others could read her thoughts (“the cashier knew how much money I had”), that colleagues communicated silently and synchronously at work, and that she might occupy a special spiritual role: “it seemed I was someone exceptional… that I was chosen.” These beliefs fluctuated in intensity over the course of her illness.

#### Phenomenology of immersive experiences

Kate’s psychopathology is characterised by auditory hallucinations accompanied by extremely vivid, often violent internal imagery. These images usually unfold within her mind, although at times they may also be experienced as visual projections in external space. During the onset of illness, for example, she experienced birds’ calls as personally directed communications and subsequently experienced an image of figures “cutting off birds’ heads and spitting them out.” More typically, however, the violent imagery remains internally located. It arises abruptly, develops rapidly, and can become highly absorbing, with a strong sense of immediacy and intense emotional and bodily effects.

When the psychiatrist-associated voice attacks her, Kate may also imagine the corresponding figure “cutting me, killing me.” She recognises these images as internally located: “I see him screaming or doing something to me… but I’ve never seen him like that in reality.” The violence often targets her own body. She described seeing “a figure coming out of me and stabbing me with a knife” or imagining the psychiatrist-associated figure “cutting off my hands.” These scenes can be accompanied by intense physical sensations: “he cuts my leg so many times that my right leg hurts terribly.” Thus, internally located imagery may be accompanied by pronounced bodily pain despite not being visually experienced in external space.

Violent imagery is also directed towards others, particularly in situations involving interpersonal conflict or frustration in her professional work. When a patient behaves rudely, mistreats a spouse, or overwhelms her with demands, Kate may feel unable to assert herself or leave the situation. At such moments, she may imagine the psychiatrist-associated figure “cutting off the patient’s leg” or killing him, sometimes followed by retaliatory actions from the dentist-associated figure. On other occasions, the scene expands to include violence directed at herself: “When I want to leave the patient’s house and I can’t say it, the voices start killing him… and then they kill me too.” These episodes are closely associated with anger, frustration, and a sense of injustice that she has difficulty expressing directly. She experiences the imagery as involuntary, unwanted, and distressing rather than as deliberately generated fantasy.

At times, Kate interpreted her voices and imagery in religious or spiritual terms. She associated the psychiatrist-related voice with a priest or spiritual authority and, during more severe episodes, feared that it might be demonic. She also described coincidences that increased her uncertainty about whether some of her internal experiences might have predictive significance. In one instance, she imagined that “something bad would happen to her [mother], that someone would smash her windows,” and shortly afterwards learned that her mother’s car had been damaged. The coincidence left her unsettled and temporarily strengthened her belief that some of her experiences might be clairvoyant.

#### Affective content

Kate’s experiences are closely associated with fear, anger, shame, and moral distress. Violent imagery often intensifies in situations of interpersonal conflict or frustration while she is providing physiotherapy to patients, particularly when she feels mistreated, overwhelmed, or unable to assert herself. She reports rarely expressing anger directly, while the accompanying imagery may involve shouting, punishment, killing, or mutilation. She experiences these scenes as frightening and morally disturbing rather than cathartic.

#### Agency and control

Kate reports having little control over the onset, content, or unfolding of her hallucinations and imagery. The violent scenes arise suddenly and develop largely independently of her intentions. Attempting to ignore or contradict the psychiatrist-associated voice may be followed by an escalation of the accompanying imagery: “if I say something in my head, he gets angry and starts hurting me.” She sometimes attempts to influence these experiences by imagining the associated figure inside a bubble or stroking it gently, but these strategies help only intermittently. She cannot determine how the violent scenes develop or end, prevent the associated figures from fighting, or stop them from attacking her. The dentist-associated figure may intervene on her behalf, but its actions are similarly outside her control.

She also uses strategies aimed at reducing the intensity of the experiences, including speaking with her mother, listening to music, relaxation exercises, and breathing techniques. She reports that these can sometimes reduce the intensity of the voices, particularly the psychiatrist-associated voice. She also speaks directly to the voices—“several times a day”—asking them to calm down or stop fighting. Although these efforts are sometimes effective, her influence over the experiences remains limited.

#### Reality attribution

Her attribution of these experiences has varied over the course of illness. During calmer periods, she recognises the voices and images as illness-related and originating from herself: “I know the voices come from me… it’s part of my illness.” During more symptomatic periods, this recognition has become less stable. She recalled earlier years when she believed the voices represented real people communicating with her, describing how she “trusted that the people were real and that they were telling the truth about me… I couldn’t tell the difference.” At times, she also attributed internally experienced communications to telepathy, believing that others could read her thoughts or communicate with her silently, and interpreted some experiences in spiritual terms. At present, she generally recognises the voices and imagery as symptoms of her illness, although she acknowledges that their emotional force can still make alternative interpretations compelling during periods of increased symptom intensity.

#### Treatment course and current status

Kate has remained in continuous psychiatric treatment since 2012, including antipsychotic medication and long-term psychotherapy. She reports that ongoing treatment, daily structure, and psychotherapy have helped her manage the experiences. Over the years, psychotherapy has included work on her relationship with the voices as well as targeted EMDR sessions focused on particularly distressing material, which she found helpful in reducing fear and increasing her ability to tolerate and reflect on these experiences.

Although auditory hallucinations and violent internal imagery persist, she has developed a clearer understanding of their triggers and more consistently recognises them as symptoms of her illness. Her daily functioning has also improved. She continues to work as a physiotherapist, uses a range of coping strategies, and maintains meaningful relationships. Despite this progress, the frequency and emotional intensity of the experiences remain a significant burden, particularly in morally charged or interpersonally stressful situations.

## Cross-case phenomenological comparison

Although all three participants described prolonged absorption in internally generated experience, comparison of the cases revealed substantial differences in agency and control, perceptual qualities, reality attribution, and psychological function. These dimensions proved more informative than the overall intensity of imaginative involvement and suggest that superficially similar experiences may reflect markedly different phenomenological configurations.

### Agency and control

The clearest differences concerned the distinction between authorship over content and control over engagement. Here, authorship refers to the person's sense of generating and shaping imagined content, rather than to the more fundamental sense of ownership of experience associated with ipseity. Disturbances of basic self-experience (Rasmussen et al., 2022; Rasmussen, 2024) were not systematically assessed in the present study. Patrick and Kate had little influence over the onset, content, or unfolding of their experiences. Their inner scenarios emerged spontaneously, developed autonomously, and frequently resisted attempts to suppress or redirect them. Both primarily experienced themselves as recipients or observers of internally unfolding events rather than as their authors. The figures represented in these experiences appeared to act independently and communicated messages that neither participant could readily influence.

Dominic’s profile differed qualitatively. His imaginative activity remained recognisably self-generated and cognitively organised. Rather than being drawn into autonomously unfolding scenes, he engaged in repetitive technical visualisation, imagining mechanical solutions and how different components might function together. His principal difficulty concerned disengagement rather than authorship: once the activity had begun, it could become prolonged, repetitive, and absorbing despite his efforts to stop or redirect attention.

### Reality attribution and perceptual qualities

The cases differed markedly in how internally generated experiences were related to reality. Patrick and Kate sometimes attributed their experiences to real agents, events, or forms of communication beyond their own imagination. Patrick believed that acquaintances, television personalities, and spiritual beings were genuinely interacting with him, while Kate at times attributed voices and imagery to real people, spiritual entities, or telepathic communication. In both cases, internally generated experiences could become incorporated into broader delusional interpretations, and these attributions varied with symptom intensity.

Their experiences also involved pronounced perceptual and bodily qualities. Patrick described vivid internally located imagery accompanied by tactile sensations, whereas Kate reported highly vivid internal imagery that could be associated with bodily pain and, at times, experienced as visual projections in external space.

Dominic’s presentation differed substantially. He consistently recognised his technical visualisations as self-generated and imaginary and did not attribute them to external events, agents, or communications. His activity could become highly absorbing, but there was no indication that the visualisations acquired comparable experiential immediacy, bodily impact, or external visual projection.

### Organisation and psychological function of imaginative experience

Although all three participants became deeply absorbed in internally generated experience, the organisation and apparent psychological functions of these experiences differed considerably. Patrick’s inner world was organised around rejection, persecution, moral worth, and spiritual conflict. Hostility and exclusion were repeatedly attributed to other people or imagined agents, which may have externalised conflict-laden evaluations of himself and his own behaviour. Kate’s imagery appeared to organise a different interpersonal conflict. Anger and aggressive impulses that she had difficulty expressing directly or using in assertive action were enacted by the figures represented in her imagery, particularly in situations in which she felt mistreated, trapped, or unable to set limits with patients. In both cases, imaginative content therefore appeared to express or organise emotionally salient conflicts, although the experiences themselves were predominantly intrusive, unwanted, and distressing rather than deliberately cultivated for pleasure or relief.

Dominic’s imaginative activity was organised differently, consisting primarily of repetitive technical visualisation of mechanical systems rather than elaborate narrative scenes involving autonomous figures. Its psychological function was more explicitly compensatory. He described using this activity to “build myself up” and escape fear of other people, while imagined technical competence and recognition temporarily countered feelings of inadequacy and loneliness. Over time, however, the same activity increasingly highlighted the discrepancy between his imagined achievements and his actual circumstances, generating frustration and regret. Its function was therefore ambivalent: it provided temporary engagement and emotional compensation while also repeatedly confronting him with unrealised aspirations.

## Discussion

The present study examined immersive imaginative experiences in three individuals with schizophrenia and compared their phenomenological structure with established descriptions of MD. Although all three participants described prolonged absorption in internally generated experience, their experiences differed substantially in authorship over content, control over engagement, perceptual qualities, reality attribution, and psychological function. Research on MD has emphasised the dynamics of engagement, including difficulty controlling or disengaging from daydreaming, as well as its rewarding and affect-regulatory functions (Greene et al., 2020; Pietkiewicz et al., 2018; Somer et al., 2017). A phenomenological perspective complements these accounts by examining the structure of imaginative experience itself. The present comparison suggests that similar levels of absorption or functional interference may occur across markedly different phenomenological configurations.

One implication concerns the frequently invoked distinction between MD and psychosis, which often rests on the premise that reality testing is preserved in MD. Existing descriptions of MD emphasise that even highly vivid and absorbing daydreams are generally recognised as self-generated and imaginary (Mansuklal et al., 2025; Somer et al., 2016). This distinction remains clinically important, but the present cases suggest that the concept of reality testing may obscure several separable aspects of experience. Recognising that an experience is internally located does not necessarily determine how real or immediate it feels while occurring, nor whether the agents or events it represents are attributed reality. Patrick, for example, could locate an encounter within his imagination while simultaneously believing that it represented a genuine interaction taking place in a spiritual reality. Dominic, by contrast, consistently recognised his technical visualisations as self-generated and imaginary. Thus, internal localisation, experiential realness or sense of presence, awareness of self-generation, and reality attribution should not be treated as interchangeable dimensions.

The distinction between MD and psychosis-related imaginative experience may therefore not be adequately captured by a binary contrast between preserved and impaired reality testing. The EAFI framework developed by Rasmussen et al. (2018), which distinguishes perceptualisation, autonomisation, and erosion of irreality, provides a useful way of decomposing this complexity into partly separable dimensions. Research in schizophrenia-spectrum disorders has also linked anomalies of imagination with disturbances of basic self-experience (*ipseity*), including the pre-reflective sense that one's experiences are one's own (Rasmussen et al., 2022; Rasmussen, 2024), although these were not systematically assessed in the present study. Perceptualisation concerns the extent to which imagery acquires quasi-perceptual qualities, whereas erosion of irreality concerns the extent to which its status as imagined remains immediately self-evident. Neither dimension is equivalent to reality attribution, which concerns whether the agents or events represented in the experience are regarded as actually existing or occurring. The sense of realness may be influenced by several interacting processes, including absorption, perceptualisation, and autonomisation, without necessarily involving the belief that imagined events are actually occurring. For research on MD, this suggests that the commonly invoked notion of preserved reality testing may be too coarse to capture potentially important phenomenological variation. Future studies may need to assess perceptualisation, experiential realness or sense of presence, awareness of self-generation, erosion of irreality, and reality attribution separately rather than subsuming them under a single judgement of whether the person “knows it is not real.”

The comparison also clarifies two distinct dimensions of impaired control over imaginative experience: authorship over content and regulation of engagement. This distinction intersects with the EAFI concept of autonomisation, which refers to the extent to which imaginative content develops its own flow relatively independently of the person’s will (Rasmussen, 2024, Rasmussen et al., 2018). Patrick and Kate showed pronounced autonomisation accompanied by markedly reduced authorship over internally unfolding content, whereas Dominic largely retained authorship but had difficulty disengaging once absorbed. Autonomisation and authorship should not, however, be treated as simple opposites: imaginative figures or sequences may acquire some independence while the person retains a broader sense of having created or shaped the imagined world. This distinction is particularly relevant to MD. Difficulty controlling daydreaming emerged as a central symptom in network analysis (Greene et al., 2020), and the proposed criteria for Daydreaming Disorder similarly emphasise difficulty resisting, limiting, or disengaging from daydreaming (Pietkiewicz et al., 2025). These formulations concern control over engagement—particularly the ability to resist, limit, or disengage from daydreaming—rather than the degree of agency a person retains over the content and unfolding of imagined experiences. Although some degree of independent agency has been described in MD characters (Soffer-Dudek & Somer, 2022), the extent and phenomenological quality of such autonomisation have not been systematically examined. Distinguishing these dimensions may help determine whether difficulty disengaging from otherwise self-authored fantasy and experiencing imaginative content as unfolding relatively independently of one’s intentions represent partly independent aspects of pathological imaginative involvement.

Absorption provides a useful point of contact between research on MD and phenomenological studies of psychosis. In research on dissociation and MD, absorption refers to intense attentional engagement in which awareness becomes narrowed around an internal or external experience (Soffer-Dudek et al., 2015), and it is strongly associated with immersive daydreaming (Somer et al., 2016). However, absorption describes the degree to which consciousness is captured rather than the phenomenological structure of the experience itself. Rosen et al. (2017) identified distinct patterns of absorption associated with attenuated versus stable ego boundaries. The present cases illustrate pronounced imaginative absorption across markedly different configurations of authorship, perceptual qualities, and reality attribution. The relationship between absorption and these phenomenological dimensions nevertheless remains unclear. Quasi-perceptual vividness or autonomous unfolding of imaginative content may facilitate deeper attentional capture, but whether greater absorption is associated with changes in other dimensions, such as perceptualisation or erosion of irreality, remains an empirical question. This distinction is also relevant to proposals to classify MD as a dissociative disorder. Although absorption and reduced awareness of the external environment form part of the phenomenological argument for such a classification (Soffer-Dudek et al., 2025), the occurrence of pronounced imaginative absorption in the present schizophrenia cases illustrates that this feature is not diagnostically specific. Absorption alone therefore provides limited grounds for determining the nosological placement of MD.

A related question concerns what makes particular imagery sufficiently significant to capture and sustain attention. In psychosis, otherwise ordinary stimuli or internal representations may acquire disproportionate significance, as described by the aberrant salience framework, which implicates dysregulated dopaminergic signalling (Kapur, 2003). Kate’s experience of bird calls as personally directed communications is compatible with this formulation. More broadly, the sources of significance that sustain immersive attention may differ across clinical contexts, raising the question of how salience, affective value, and absorption are interrelated. Preliminary pharmacological observations raise questions about possible medication-related changes in pathological daydreaming outside psychosis. Mamah et al. (2025) reported rapid reduction or cessation of daydreaming during treatment with lurasidone, risperidone, and aripiprazole, whereas lisdexamfetamine and bupropion were associated with substantially increased time spent daydreaming. In the present study, Dominic similarly reported a marked reduction in prolonged imaginative activity after the introduction of lurasidone, whereas treatment with amisulpride and aripiprazole had not produced a comparable effect. These observations cannot establish a causal relationship or identify the mechanisms responsible for the reported changes, but warrant further investigation.

Content, emotional valence, and psychological function provide another level of description, but none appears diagnostically specific in isolation. In a large screening-based study, individuals who self-identified as maladaptive daydreamers and scored above the cutoff on an MD screening measure reported a wide range of fantasy themes, including morbid and violent imagery and trauma-related reenactments (Somer et al., 2021). However, participants were not clinically assessed to establish whether all reported internal experiences represented the same phenomenological form of daydreaming or to differentiate them systematically from rumination, trauma-related re-experiencing, intrusive imagery, or other forms of repetitive internal mentation. These findings therefore demonstrate the range of contents reported by individuals screening positive for MD, but do not establish that every such experience belongs to the core MD phenotype. Importantly, emotional valence should not be equated with psychological function. In the present cases, Patrick’s and Kate’s imagery was predominantly intrusive and distressing, yet its content appeared to organise or express emotionally salient conflicts, whereas Dominic’s imaginative activity was initially compensatory and engaging but became increasingly frustrating as it highlighted the discrepancy between imagined competence and his actual circumstances. Interestingly, shortly after lurasidone markedly reduced his imaginative activity, Dominic asked for the medication to be discontinued because he did not want to lose the capacity to fantasise.

These considerations caution against defining pathological daydreaming by either the pleasantness of its content or its affect-regulatory function. Clarifying the MD phenotype requires first determining whether the mental activity takes the form of elaborated imagined scenes, interactions, or narratives rather than rumination, trauma-related re-experiencing, ordinary problem-solving, or unstructured mind-wandering. This distinction may not be sufficient, as elaborate internal scenarios are not specific to MD. In DID, for example, a person may experience internally unfolding interactions among dissociative self-states as an observer rather than as the author of the scene. Assessment of agency and authorship may therefore provide an additional differential dimension: how much the person retains a sense of generating and shaping the imagined world. Only after these phenomenological boundaries are established does it become meaningful to examine whether the activity shows the impaired control, increasing priority, and persistence despite harm proposed as defining features of Daydreaming Disorder (Pietkiewicz et al., 2025).

A further differential dimension concerns how imaginative experience relates to other psychopathological phenomena. In Patrick and Kate, internally generated imagery became intertwined with voices, bodily sensations, ideas of reference, and delusional interpretations. The present material cannot establish whether imagery contributed to the development or strengthening of delusional beliefs or vice versa, or whether these processes developed reciprocally. For research on MD, this highlights the importance of examining not only the phenomenological characteristics of daydreaming but also its relationship to concurrent beliefs, hallucinations, and other psychopathological phenomena (Raballo, 2017).

Dominic raises a different question about the boundaries of the MD phenotype. His imaginative activity was prolonged, difficult to disengage from, increasingly prioritised, and associated with functional interference, yet its predominant form differed markedly from the elaborate imagined scenes, interactions, and narratives characteristic of daydreaming. Instead, it consisted primarily of repetitive technical visualisation involving the imagined design and manipulation of mechanical systems, rather than extended narrative fantasy. His case therefore highlights the importance of establishing that the target mental activity falls within the phenomenological domain of daydreaming before evaluating the core mechanisms proposed for Daydreaming Disorder. This distinction may also be relevant in ASD, where intense repetitive interests and distinctive patterns of mental imagery may coexist with, but should not be equated with, immersive daydreaming (Bled et al., 2024; West et al., 2023). His case therefore highlights the importance of clearly defining the target phenomenology of daydreaming to which the proposed Daydreaming Disorder criteria are applied.

Taken together, the cases support a configurational rather than feature-based approach to pathological imaginative involvement. Imaginative absorption, vividness, impaired disengagement, autonomous content, emotional involvement, and functional interference may each occur across different clinical contexts. Their diagnostic significance may therefore depend on how they are organised together—for example, whether imaginative content is experienced as authored or autonomous, how engagement is regulated, whether represented agents or events are attributed reality, and how the experience relates to other psychopathological phenomena. Such a framework may help distinguish MD from other forms of persistent internally generated experience without assuming that any single feature is diagnostically specific.

## Clinical implications

The findings highlight the importance of detailed phenomenological assessment when patients describe immersive fantasy, imagery, daydreaming, or “inner worlds,” as these terms may refer to substantially different forms of internal experience. Assessment should clarify authorship and autonomisation of content, regulation of engagement, perceptual qualities, reality attribution, and the relationship of these experiences to hallucinations, delusional beliefs, identity phenomena, or other psychopathology. This is particularly important when interpreting MD screening measures in clinical populations, where high scores may reflect superficially similar but phenomenologically distinct forms of persistent internal experience.

## Limitations and future directions

Several limitations should be considered. First, this was a deliberately idiographic case series of three purposively selected individuals with schizophrenia who reported pronounced imaginative absorption. The cases were intended to expose clinically relevant phenomenological differences rather than establish their frequency or typicality; the findings should therefore not be generalised to schizophrenia more broadly. Second, MD was not examined in a directly recruited comparison group, and comparisons relied on phenomenological descriptions in the existing literature. Proposed differences between MD and psychosis-related imaginative experience therefore remain provisional until the same assessment framework is applied directly across clinical groups. The temporal relationship between interviews and the reported phenomena also differed across participants: Patrick and Dominic described important aspects retrospectively, whereas Kate continued to experience psychotic symptoms during data collection. Retrospective accounts may have been shaped by subsequent insight and reconstruction, while ongoing symptoms may have influenced how experiences were described and interpreted at interview.

Third, the data were generated in a clinical context and analysed using theory-informed dimensions derived from prior work on MD and schizophrenia-spectrum psychopathology. The interviews explored the content, emotional significance, psychological functions, and regulation of imaginative activity, alongside its phenomenological characteristics. Although several domains overlapped with those addressed by the EAFI, the instrument was not administered systematically, potentially limiting the depth of exploration of certain structural anomalies of imagination. Longstanding clinical contact provided access to detailed and longitudinal material but may also have influenced how experiences were narrated and interpreted. The proposed phenomenological distinctions therefore require further examination using more standardised assessment procedures and independent coding. Finally, ASD was not systematically assessed in Dominic, leaving the possible relevance of autism-related cognitive style or imagery uncertain. Patrick’s early illness developed against a background of extensive substance use, including cannabis, stimulants, and hallucinogens, which complicates interpretation of his early anomalous experiences. Although his psychotic symptoms persisted outside intoxication and schizophrenia was subsequently diagnosed, the contribution of substance exposure cannot be determined from the present data.

Future research should apply the same phenomenological framework directly across MD and other psychiatric disorders to test whether dimensions often subsumed under broad constructs such as “sense of reality” or “impaired control” can be empirically distinguished. Perceptualisation, experiential presence, awareness of self-generation, erosion of irreality, reality attribution, autonomisation, and regulation of engagement should be assessed separately to determine which dimensions covary, which vary independently, and whether different configurations characterise different forms of pathological imaginative involvement. Their relationships with absorption should also be examined, rather than assuming that greater absorption entails a particular phenomenological configuration.

A further question concerns what makes particular internally generated representations sufficiently compelling to capture and sustain attention. Comparative research could examine how salience, affective significance, and reward relate to prolonged imaginative engagement across MD and other psychiatric disorders, and whether similar patterns of persistence or impaired control arise from different underlying processes. Longitudinal and experience-sampling designs may also help clarify how imaginative activity, affect, hallucinations, and delusional interpretations influence one another over time.

## Conclusion

The present cases suggest that prolonged imaginative absorption is neither specific to MD nor phenomenologically uniform. Experiences that appear similar in their intensity, persistence, or difficulty of control may differ substantially in authorship, perceptualisation, reality attribution, and their relationship to other psychopathological phenomena. A phenomenological approach therefore complements behavioural and functional accounts of MD by emphasising the configuration of features rather than any single characteristic of imaginative involvement. Comparative research using the same framework across MD and other psychiatric disorders may help clarify the boundaries of the Daydreaming Disorder phenotype and distinguish its defining features from broader transdiagnostic processes.

## Funding

This research did not receive any specific grant from funding agencies in the public, commercial, or not-for-profit sectors.

## Declaration of generative AI and AI-assisted technologies

During the preparation of this work, the authors used ChatGPT (OpenAI) to assist with language editing and improve the clarity and readability of the manuscript. After using this tool, the authors reviewed and edited the content as needed and take full responsibility for the content of the published article.

## Declaration of competing interest

The authors declare that they have no known competing financial interests or personal relationships that could have appeared to influence the work reported in this paper.

## Data Availability

The interview transcripts contain sensitive and confidential clinical information and are therefore not publicly available in order to protect participants’ privacy. The data are securely retained by the authors in accordance with applicable legal and ethical requirements and the conditions of the ethics approval.
